# Meckel's Diverticulum Enterolith: A Rare Cause of Perforation and Small Bowel Obstruction Presenting as Acute Abdomen

**DOI:** 10.7759/cureus.20363

**Published:** 2021-12-12

**Authors:** Ahmad LF Yasin, Amal MJ Thabet, Amna Sadiq, Ahmad Hisham Mohammad Shaban, Ali Toffaha, Aalaa Kambal

**Affiliations:** 1 Radiology, Hamad Medical Corporation, Doha, QAT; 2 Clinical Attachment, Radiology, Hamad Medical Corporation, Doha, QAT; 3 General Surgery, Hamad Medical Corporation, Doha, QAT

**Keywords:** phlegmon, intestinal obstruction, perforation, enterolith, meckel’s diverticulum

## Abstract

Meckel’s diverticulum (MD) is the most common congenital anomaly of the gastrointestinal tract. MD enteroliths are an uncommon entity that could rarely present with complications.

We, herein, present a case of a 56-year-old man who presented with severe abdominal pain. Preoperative CT showed an inflamed structure with central calcification connected to distal ileum lumen with an air pouch associated with small bowel obstruction. Upon laparotomy, a perforated MD was found with phlegmon formation. He underwent resection of the loops containing MD with end-to-end anastomosis.

This highlights the importance of radiological imaging in preoperative diagnosis of these conditions which should be kept in mind if we find a calcified lesion in an inflamed diverticulum around the midline region.

## Introduction

Meckel's diverticulum (MD) is considered as the most common congenital anomaly of the gastrointestinal tract. Most patients with MD remain clinically silent [1]. They become symptomatic when MD is complicated by bleeding, obstruction, or inflammation [2]. Small bowel obstruction happens when Meckel’s diverticulum (MD) is complicated by intussusception, adhesions, volvulus, neoplasm, or the formation of enteroliths [3]. We report a rare case of Meckel’s enterolith that led to perforation and bowel obstruction.

## Case presentation

A 56-year-old Asian male presented to the emergency department (ED) with three days history of diffuse abdominal pain which was more severe in the periumbilical region. The pain was associated with high-grade fever and vomiting. There was no history of diarrhea or constipation. He had a history of appendectomy 40 years ago.

On physical examination, the patient's vital signs were stable apart from fever. The abdomen was distended, and there was tenderness and guarding around the umbilical area. There were no abdominal palpable masses. Cardiac, respiratory, and neurological examinations were unremarkable.

**Figure 1:**
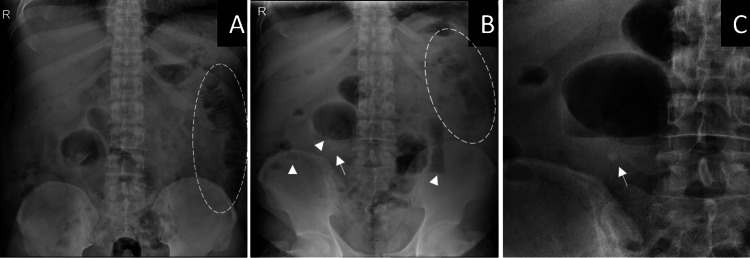
Abdominal radiograph. Supine (A) and erect (B) X-ray views show signs of small bowel obstruction including dilated small bowels with visible valvulae conniventes (dashed circles) and few air fluid levels (arrow heads). Small rounded calcified density is seen in the right lower quadrant (arrow) which is better visualized in the magnified view (C).

Laboratory investigations revealed a high white cell count (15 x 106/L) and C-reactive protein (320 mg/L). The rest of the laboratory results were unremarkable. Abdominal X-ray revealed dilated small bowel loops with few air-fluid levels (Figure 1) which suggested small bowel obstruction. Small calcification was noted in the right lower quadrant.

**Figure 2:**
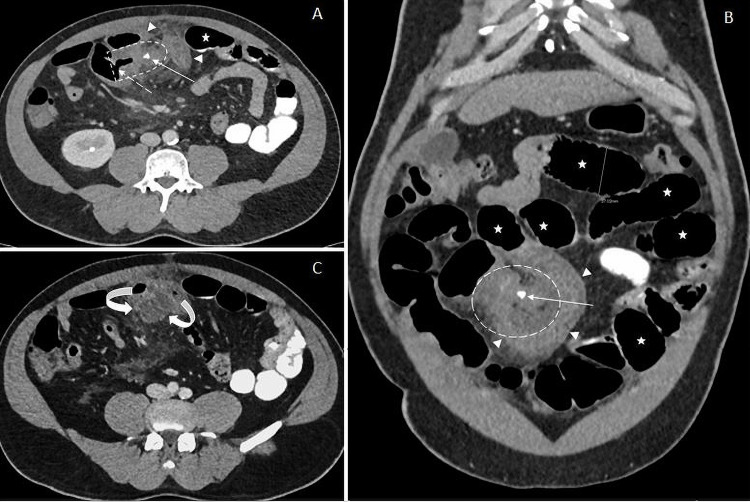
Contrast-enhanced abdominal CT scan. Axial (A) and coronal (B) images show multiple dilated small bowel loops (stars). Short segment of terminal ileum showing narrowed lumen and thickened edematous wall (arrow heads). It forms a U-shaped loop around an ill-defined soft tissue enhancing structure (dashed circle) with central dense focal calcification (arrow) and surrounded by extensive fat stranding and traces of free fluid within the mesentery. This structure is inseparable from the distal part of the ileum and is connected to its lumen through air containing pouch (dashed arrow). Caudal axial section (C) shows small fluid collection (curved arrows) with severe mesenteric fat stranding. The expected shape of the obstructed MD is denoted in image A (dashed delay shape). MD, Meckel's diverticulum

Then the patient had contrast-enhanced CT of the abdomen (Figure 2) which also showed multiple dilated small bowel loops in the abdomen including the jejunum and proximal ileum. The CT scan also showed a short segment of the ileum that demonstrated narrowed lumen and thickened edematous wall that corresponded to the transition point. It formed a U-shaped loop around an ill-defined enhancing structure with central dense focal calcification and surrounded by extensive fat stranding and traces of free fluid within the mesentery. This structure is inseparable from the distal part of the ileum and is connected to its lumen through air containing pouch. No definite pneumoperitoneum was noted.

The patient had exploratory laparotomy. Operative findings included purulent peritonitis and a mass-like structure originating from the anti-mesenteric border of the mid ileum with adhesions of multiple loops of ileum. Adhesions were released and the patient underwent small bowel resection of the loops containing this inflammatory structure with subsequent end-to-end anastomosis. Histopathology of the specimen revealed severely inflamed and ruptured MD. The patient had an uncomplicated post-operative course and recovered successfully.

## Discussion

Meckel's diverticulum is a rare congenital anomaly. It is a remnant of the incompletely obliterated omphalomesenteric or vitelline duct; it is reported as a true diverticulum because it has the whole layers of the intestinal wall [4]. The vast majority of patients with MD remain clinically asymptomatic [5]. Only 16%-20% of MD patients develop symptoms that occur when complicated. The most common clinical presentations of complicated MD are intestinal hemorrhage, bowel obstruction, and inflammation [1]. In children, painless intestinal hemorrhage and obstruction are the most common complaints, while obstructive complaints are prevalent among adults [6].

In the case of MD, enteroliths are developed only in 3%-10% of the patients with the most reported cases occurring in males. Multiple theories were reported about the factors predisposing to enteroliths formation including inflammation, intestinal stasis, and severe intraluminal alkalotic or acidotic environment [7]. The presence of enterolith in association with MD might lead to obstructed bowels due to inflammation, outer compression of other loops, intussusception, or lodging of enterolith into distal bowel after expulsion from the diverticulum [8]. MD perforation is one of the most severe complications and can happen as a result of gangrene, diverticulitis, peptic ulcer, or rarely as in our case due to an enterolith. The explanation of reporting only a few cases of perforated MD in the case of true stone refers to the fact that most MDs have a wide neck and peristaltic smooth muscles making it difficult for the MD to be obstructed. Misdiagnosis of perforated MD associated with enterolith is not uncommon and is challenging due to the similarities with other conditions of the acute abdomen [9]. Differential diagnosis includes mesenteric carcinoid tumor, calcified lymph node, gallstone ileus, urinary calculi, swallowed foreign body, appendicolith, or teratomas [2].

The knowledge of the clinical presentation, complications, and imaging findings of MD anomaly can assist in the definitive diagnosis and management [8]. It should be included in the differential diagnosis especially when radiological studies report a calcified lesion preoperatively as 30%-50% of these enteroliths are radio-opaque and both abdominal radiograph and CT scan can frequently show them [3]. Typical findings include a rounded or triangular radio-opaque lesion that shows a hyperdense periphery with central radiolucency located in the lower abdomen [7]. CT scan has been stated as the first imaging modality in the diagnosis of any surgical and inflammatory abdominal conditions; for MD the sensitivity of diagnosis has increased with the development of higher spatial resolution and multiplanes reconstructing CT scanners [5].

Our case demonstrated a diagnostic challenge as the extensive inflammation and adhesions obscured the normal outline and appearance of the MD. The presence of a triangular calcified lesion within an inflamed structure that is connected to the ileum with an air-containing pouch was the basis on which the diagnosis of MD with enterolith was on top among other differential diagnoses. In addition to that, the presence of severe surrounding inflammatory changes with phlegmon formation involving the mesentery and adjacent loops of bowel suggested a perforated MD with adhesions involving the surrounding bowels that led to intestinal obstruction.

Bowel perforation should not usually result in pneumoperitoneum, but the leakage of liquid contents from the bowel may form abscesses or phlegmon [10].

Despite the classical evidence which stated that surgery is the gold standard for diagnosis of MD and its complications, our case showed that the radiological findings which could be preceding in suggesting the diagnosis as intraoperative findings, in this case, were not highly conclusive due to severe inflammation in the operative field and the diagnosis was only confirmed only by histopathology.

Exploratory laparotomy is the safest and most accurate modality in the diagnosis and treatment of both symptomatic and complicated MD. There are several operative options including milking or fragmentation of the enterolith, creating an enterotomy followed by enterolith extraction or resection of the segment of the small bowel containing the inflamed MD and enterolith followed by primary anastomosis, which was performed in our case [1, 8].

## Conclusions

Enterolith of MD causing perforation and small bowel obstruction is extremely rare and poses a diagnostic challenge. When a calcified density is detected in the lower abdomen particularly around the midline, MD enterolith should be kept in the differential diagnosis particularly in the presence of signs of inflamed diverticula which is denoted by a direct connection to the small bowel lumen.
